# The Between-Day Reliability of Correlation Properties of Heart Rate Variability During Running

**DOI:** 10.1007/s10484-023-09599-x

**Published:** 2023-07-29

**Authors:** Bas Van Hooren, Bart C. Bongers, Bruce Rogers, Thomas Gronwald

**Affiliations:** 1 Department of Nutrition and Movement Sciences, School of Nutrition and Translational Research in Metabolism (NUTRIM), Faculty of Health, Medicine and Life Sciences, Maastricht University, Universiteitssingel 50, 6229 ER Maastricht, The Netherlands; 2 Department of Surgery, School of Nutrition and Translational Research in Metabolism (NUTRIM), Faculty of Health, Medicine and Life Sciences, Maastricht University, Maastricht, The Netherlands; 3 College of Medicine, University of Central Florida, Orlando, FL USA; 4 Institute of Interdisciplinary Exercise Science and Sports Medicine, MSH Medical School Hamburg, Hamburg, Germany

**Keywords:** HRV, Detrended fluctuation analysis, DFA a1, Endurance exercise, Reliability

## Abstract

The short-term scaling exponent of detrended fluctuation analysis (DFA-a1) of heart rate variability may be a helpful tool to assess autonomic balance as a prelude to daily, individualized training. For this concept to be useful, between-session reliability should be acceptable. The aim of this study was to explore the reliability of DFA-a1 during a low-intensity exercise session in both a non-fatigued and a fatigued condition in healthy males and females. Ten participants completed two sessions with each containing an exhaustive treadmill ramp protocol. Before and after the fatiguing ramp, a standardized submaximal low-intensity exercise bout was performed during which DFA-a1, heart rate, and oxygen consumption (VO_2_) were measured. We compared between-session reliability of all metrics prior to the ramps (i.e., non-fatigued status) and after the first ramp (i.e., fatigued status). Intraclass correlation coefficients (ICC) with 95% confidence intervals (CI), the standard error of measurement, and the smallest worthwhile change (SWC) were determined. The ICC and SWC pre fatiguing ramp were 0.85 (95% CI 0.39–0.96) and 5.5% for DFA-a1, 0.85 (0.38–0.96) and 2.2% for heart rate, and 0.84 (0.31–0.96) and 3.1% for VO_2_. Post fatiguing ramp, the ICC and SWC were 0.55 (0.00–0.89) and 7.9% for DFA-a1, 0.91 (0.62–0.98) and 1.6% for heart rate, and 0.80 (0.17–0.95) and 3.0% for VO_2_. DFA-a1 shows generally acceptable to good between-session reliability with a SWC of 0.06 and 0.07 (5.5–7.9%) during non-fatigued and fatigued conditions. This suggests that this metric may be useful to inform on training readiness.

## Introduction

An adequate balance between physical exercise training load and recovery is required to induce optimal adaptations to endurance training. The ideal training load (i.e., frequency, intensity, and time) for a given training session is however difficult to prescribe due to limitations in the metrics used to assess athletes’ physiological status or fatigue. For example, while there are several indices or biomarkers that have been shown sensitive to fatigue such as variables collected during countermovement jumps or drop jumps, muscle enzyme concentrations (e.g., creatine phosphokinase), and salivary hormones (Rogers & Gronwald, 2022), these metrics are typically collected after training or during rest periods and are therefore not helpful to assess fatigue during endurance training sessions or competition. Further, some metrics reflect local rather than whole body fatigue, and their validity can be challenged by contextual and individual factors (e.g., motivation, familiarization, physical qualities, seasonal stage) (Shushan et al., 2022). Finally, because the sensitivity of these metrics depends on the time of assessment (Guthrie et al., 2022), decision-making based solely on (neuromuscular) performance factors is likely suboptimal.

Heart rate (HR) as regulated by the autonomic nervous system may be used as a proxy of whole-body fatigue during physical exercise training sessions and thereby overcomes several of the limitations of other methods. However, the validity of HR as a proxy of fatigue has been questioned, for example due to effect of HR (cardiac) drift or the indication of opposing trends in adaptation processes (Mattsson et al., 2011; Maunder et al., 2021; Schimpchen et al., 2023). The variability of cardiac beat-to-beat intervals (i.e., heart rate variability; HRV) may be more useful to inform on the degree of autonomous fatigue during endurance training or competition. Specifically, a non-linear HRV index based on fractal correlation properties, termed alpha1 (short-term scaling exponent) of detrended fluctuation analysis (DFA-a1), has been shown to have utility as a marker of fatigue in recent studies (Gronwald et al., 2019, 2021; Rogers & Gronwald, 2022; Rogers et al., 2021b). For example, DFA-a1 has been shown to be significantly lower after a 6-h simulated ultramarathon when running at a speed close to the first ventilatory threshold (Rogers et al., 2021b). Similarly, during a marathon race, DFA-a1 decreased from 0.54 to 0.37 despite significant increases in km split times (Gronwald et al., 2021). Finally, DFA-a1 was also markedly suppressed at the first ventilatory threshold when assessed during a second incremental test (Van Hooren et al., 2022).

In addition as a marker of fatigue, DFA-a1 may also be useful to inform on the physiological status of an athlete as a surrogate of daily directed training or “training readiness”. Sample case data and data of a pilot study with decreased DFA-a1 values during a standardized warm-up prior to a planned training session lead to the hypotheses that this metric could be used for decision support to modify the training load of the session to avoid excess training load (Rogers & Gronwald, 2022; Schaffarczyk et al., 2022). In support of this, recent studies have shown that adjustments of training intensity based on resting HRV leads to larger improvements in physiological parameters and performance than pre-defined training (Duking et al., 2021; Nuuttila et al., 2022). Although it remains to be proven whether the assessment of DFA-a1 during a standardized low-intensity exercise session may have similar benefits, a first prerequisite is that this metric should show adequate reliability between sessions so that potential small changes due to fatigue can accurately be detected. Since the between-day reliability of DFA-a1 has not yet been investigated, the aim of the present study was to explore the reliability of DFA-a1 during a low intensity physical exercise training session in a non-fatigued and a fatigued condition in healthy males and females.

## Methods

### Experimental Approach

This study involved two experimental sessions separated by approximately one week. The data reported here was collected as part of a study that aimed to investigate the effect of fatigue on DFA-a1 behavior (Van Hooren et al., 2022). During each session, each participant completed an incremental running test with a standardized low intensity run prior and post the incremental test while HRV and respiratory gas exchange data were recorded simultaneously. Participants were instructed to avoid strenuous activity for 36 h, alcohol for 24 h, caffeine for 6 h, and a heavy meal 2 h before the session. The study was approved by the local ethics committee (reference FHML-REC/2021/078), and all participants were informed about the study’s context, voluntary nature, procedures, benefits, and potential risks, and signed an informed consent form prior to the measurements.

### Participants

Ten participants (8 males, 2 females; mean ± SD: age 22.9 ± 2.2 years; body mass 75.7 ± 12.7 kg; body height 178.7 ± 9.5 cm), that were free of any moderate (for previous 3 months) or minor (for previous 1 month) musculoskeletal injuries, were not taking any medication that could influence running performance, were aged 18–45 years, comfortable with treadmill running, and had a body mass index (BMI) < 30 kg/m^2^ volunteered to participate in this study.

### Exercise Protocol

Each session consisted of an exhaustive ramp protocol performed on a treadmill (Technogym, Excite 700, Italy) to elicit a maximal effort. Before and after the ramp a standardized submaximal low-intensity exercise bout was performed.

**Fig. 1:**
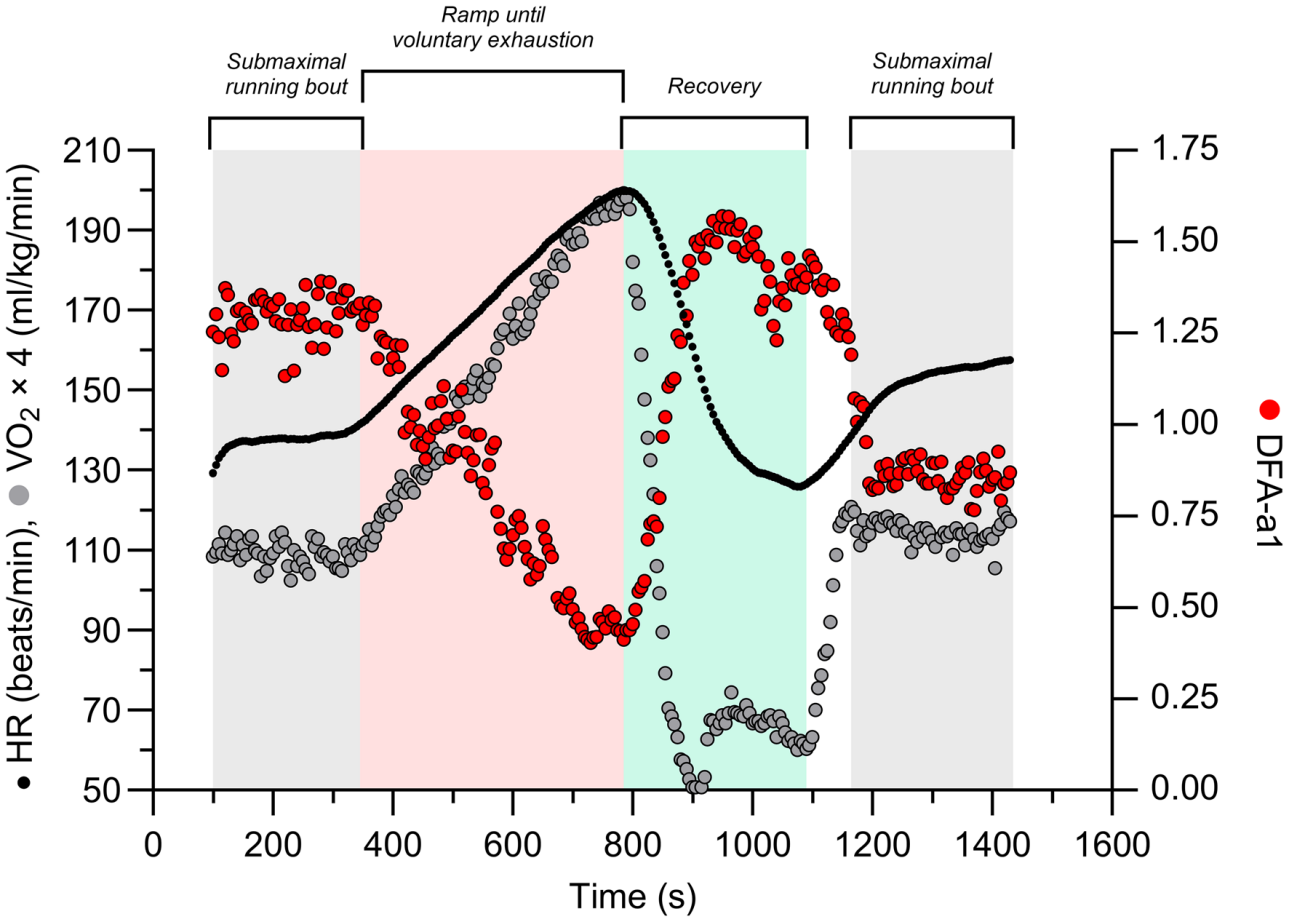
Example of submaximal running bouts at the same running speed (7–9 km h^−1^: based on heart rate reserve) before and after the ramp protocol until voluntary exhaustion and a short recovery period; alpha1 of detrended fluctuation analysis (DFA-a1), heart rate, and oxygen consumption (VO_2_) data of one participant displayed over time

The protocol started with 5 min of submaximal running at 7–9 km h^−1^ depending on whether the HR was < 35–45% of the HR reserve (calculated by subtracting the resting HR from the estimated maximum HR [using the Tanaka equation (Tanaka et al., 2001)], multiplying the result with 0.4 and then adding this to the resting HR). The ramp protocol consisted of increments of 0.5 km h^−1^ every 30 s until a speed of 20 km h^−1^ was reached. Thereafter, treadmill incline was increased by 1° every 30 s. The ramp ended when the participant reached volitional exhaustion. The participant then rested for 5 min, of which the first 2 min were passive rest (standing), and the other 3 min involved slow walking on the treadmill. After the 5-min rest period, a second submaximal protocol started at the same speed as the first 5 min to assess the effect of residual fatigue from the ramp test on reliability (see Fig. 1). A fan was placed 1 m in front of the treadmill to maintain a cool body temperature and mimic air flow to maximize transfer to outdoor conditions.

### Equipment

Participants wore a face mark (Hans Rudolph Inc, Shawnee, KS, USA) over the nose and mouth without detectable leakage to collect respiratory gasses throughout all test conditions. The mask was connected to a T-piece that was placed in a free airstream (200 L min^–1^). Respiratory gases were measured continuously and computed at 5-s intervals using an indirect calorimeter (Omnical v6, Maastricht Instruments, Maastricht, The Netherlands). The system was calibrated automatically every 15–30 min using room air and a gas mixture of known composition.

HR and HRV were recorded using a chest belt (Polar H10, Polar Electro Oy, Kempele, Finland) with a sampling rate of 1000 Hz that was tightly secured by the researchers. RR-interval data was sent to an open-source mobile application (FatMaxxer, https://github.com/IanPeake/FatMaxxer) via Bluetooth where it was monitored in real-time and saved for further analyses. RR recordings and ECG tracings (automatically taken during detected artifact) of the incremental test were visually inspected to ensure proper quality, checking for missed beat artifact, and/or noise and arrhythmia during each measurement.

### Data Processing

For analysis of the standardized submaximal exercise bouts prior and post the ramp until voluntary exhaustion, respiratory gas exchange data was imported into Microsoft Excel 365. In addition, RR data was exported as a text file for analysis in Kubios HRV Premium Software Version 3.5 for analyzing HR and HRV. Preprocess settings were at the default values and RR detrending method was at “Smoothness priors” (Lambda = 500). RR-interval series were corrected by the Kubios “automatic threshold”. DFA-a1 window width was defined to 4 ≤ n ≤ 16 beats. Before analysis, all tachograms were inspected to distinguish and correct artefacts. To minimize DFA-a1 bias, the acceptable limit of participant-related artifact was kept at or below 3% (Rogers et al., 2020; Van Hooren et al., 2022). The Kubios HRV data output (HR and DFA-a1) was time-aligned with the Omnical-based gas exchange data (e.g., oxygen consumption (VO_2_) and analyzed during a 2-min window (minute 3 and 4 of the 5-min submaximal exercise bouts). Rating of perceived exertion (RPE), respiratory exchange ratio (RER), VO_2peak_ and maximum HR during the exercise ramp test was also determined (VO_2peak_ as a proxy of cardiorespiratory fitness) and calculated as the highest moving average value obtained over 30 consecutive seconds.

### Statistical Analyses

Statistical analyses were done using SPSS 25.0 (IBM Statistics, United States) for Windows (Microsoft, USA). Normality was assessed using visual inspection of Q–Q plots and histograms. DFA-a1, HR, and VO_2_ during the submaximal and maximal exercise bout(s) were presented as mean and standard deviations. For the maximal ramp test, the RER and RPE at peak exercise were also reported. Relative reliability between the 2 days (PRE1 vs. PRE2; peak1 vs. peak2; POST2 vs. POST2) was evaluated using a mean rating two-way random model intraclass correlation coefficient (ICC) for absolute agreement. This ICC model was chosen to allow generalization beyond the current experiments, and to incorporate both systematic and random sources of error. The 95% confidence intervals (CI) were also computed. The ICC was interpreted as poor: < 0.5, moderate: ≥ 0.5 to < 0.75, good: ≥ 0.75 to < 0.9, or excellent: ≥ 0.9 (Koo & Li, 2016).

The standard error of measurement (SEM; also described as typical error, TE (Hopkins, 2000) was determined by calculating the standard deviation of the difference scores between the trials on each day and dividing this by the square root of two (Hopkins, 2000; Swinton et al., 2018). The SEM was expressed in original and percentage units (i.e., coefficient of variation; CV), with the latter one determined by dividing the SEM in original units by the grand mean of both measurements multiplied by 100. The smallest worthwhile change (SWC) was computed as 0.5 × CV (Buchheit, 2014).

## Results

Descriptive and reliability statistics for both test days are reported in Table 1. Briefly, the ICC and SWC pre fatiguing ramp were 0.85 (95% CI 0.39–0.96) and 5.5% for DFA-a1, 0.85 (0.38–0.96) and 2.2% for HR, and 0.84 (0.31–0.96) and 3.1% for VO_2_. Post fatiguing ramp, the ICC and SWC were 0.55 (0.0–0.89) and 7.9% for DFA-a1, 0.91 (0.62–0.98) and 1.6% for HR, and 0.80 (0.17–0.95) and 3.0% for VO_2_.

**Table 1:** Descriptive statistics and reliability statistics for both test days

Variable	Mean ± SD 1st day	Mean ± SD 2nd day	Mean ICC (95% CI)	Mean SEM in original and percentage units (CV)	Mean SWC in original and percentage units
*Low-intensity pre ramp*					
HR (beats min^−1^)	141 ± 13.1	140 ± 10.5	0.85 (0.38–0.96)	6.23 (4.42%)	3.11 (2.21%)
VO_2_ (ml kg^−1^ min^−1^)	30.3 ± 3.6	30.1 ± 3.2	0.84 (0.31–0.96)	1.88 (6.24%)	0.94 (3.12%)
DFA-a1	1.12 ± 0.23	1.12 ± 0.24	0.85 (0.39–0.96)	0.12 (11.0%)	0.06 (5.49%)
*Peak during ramp*					
HR_peak_ (beats min^−1^)	193 ± 6.0	193 ± 5.1	0.93 (0.70–0.98)	2.16 (1.12%)	1.08 (0.56%)
VO_2peak_ (ml kg^−1^ min^−1^)	55.0 ± 9.0	53.8 ± 9.0	0.99 (0.93–0.99)	1.04 (1.91%)	0.52 (0.96%)
RER_peak_	1.16 ± 0.40	1.14 ± 0.36	0.77 (0.16–0.94)	0.02 (1.93%)	0.01 (0.96%)
RPE	17.6 ± 1.7	17.4 ± 1.6	0.81 (0.21–0.95)	0.97 (5.57%)	0.49 (2.78%)
*Low-intensity post ramp*					
HR (beats min^−1^)	153 ± 13.1	154 ± 10.0	0.91 (0.62–0.98)	5.03 (3.27%)	2.51 (1.64%)
VO_2_ (ml kg^−1^ min^−1^)	31.9 ± 3.3	31.5 ± 3.2	0.80 (0.17–0.95)	1.91 (6.03%)	0.96 (3.01%)
DFA-a1	0.87 ± 0.20	0.88 ± 0.14	0.55 (0.00–0.89)	0.14 (15.8%)	0.07 (7.92%)

*CI *confidence interval, *DFA-a1 *alpha1 of detrended fluctuation analysis, *HR *heart rate, *HR*_peak_ heart rate at peak exercise, *ICC* intraclass correlation coefficient, *RER*_peak_ respiratory exchange ratio at peak exercise, *SD* standard deviation, *SEM* standard error of measurement, *SWC* smallest worthwhile change, *VO*_*2*_ rate of oxygen consumption, *VO*_*2*peak_ rate of oxygen consumption at peak exercise

## Discussion

The aim of this brief report was to explore the between-day reliability of DFA-a1 during a low intensity physical exercise training session in a non-fatigued and a fatigued condition in healthy males and females. Sample case and pilot studies have suggested that measurement of the DFA-a1 value during a standardized warm-up prior to a planned training session may be used to inform generally on the physiological status and specifically on autonomic fatigue, which in turn may be used for decision-support to modify the training load of the upcoming session (Rogers & Gronwald, 2022; Schaffarczyk et al., 2022). However, when adopting such an approach, researchers and practitioners should first know the variation that can be expected to occur in DFA-a1 simply due to technical measurement error and biological variability independent of autonomic fatigue. To investigate this ‘typical variation’, we had participants perform a low-intensity session at two different days and assessed various physiological metrics to quantify between-day reliability. Our results show that the DFA-a1 value exhibited good relative reliability (e.g., an individual with a high DFA-a1 relative to the other individuals in session one generally also exhibited a high DFA-a1 value relative to the other individuals in session two) prior to the fatiguing ramp, and this reliability was comparable to the relative reliability for HR and VO_2_ (Table 1). However, the absolute reliability as assessed by the CV was lower for DFA-a1 than for HR and VO_2_. It followed from this that the smallest worthwhile change was also larger. For example, the SWC pre ramp for DFA-a1 was 5.5% as compared to 2.2% and 3.1% for HR and VO_2_, respectively.

**Fig. 2:**
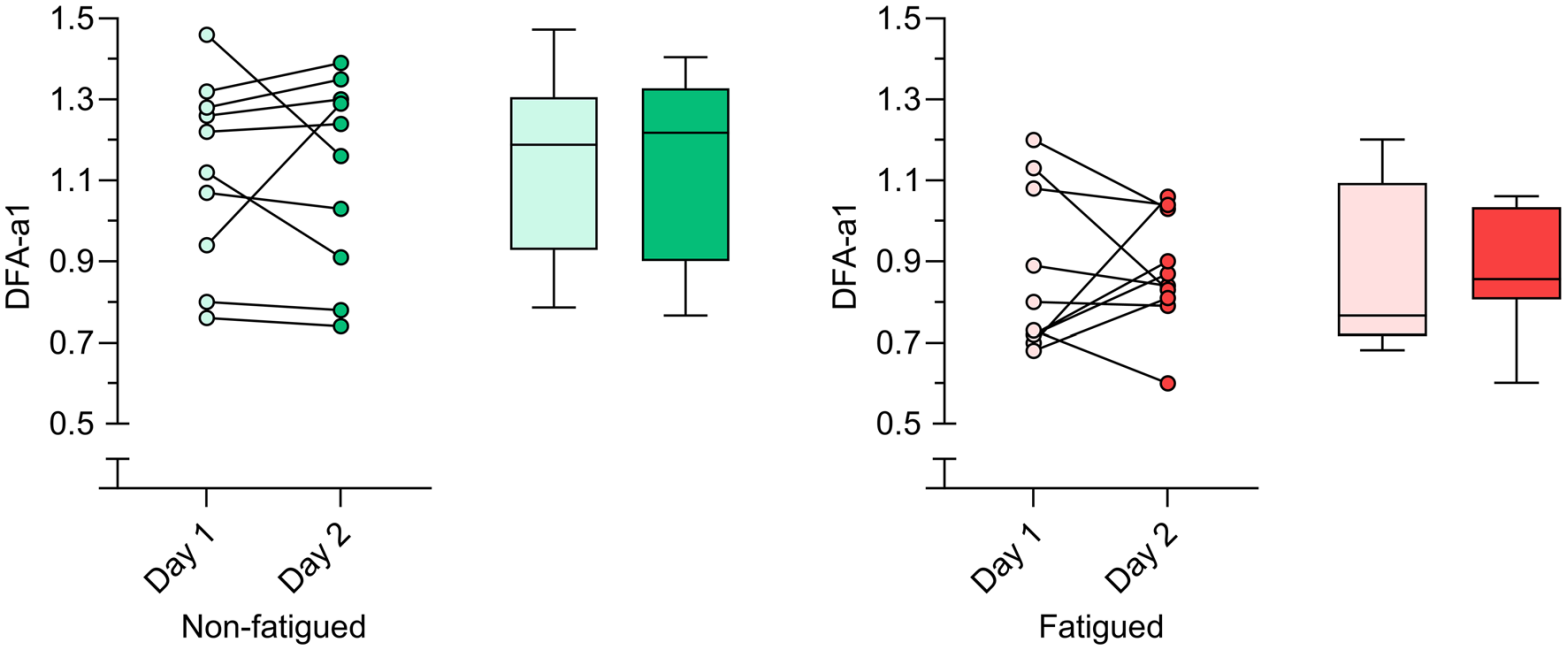
Change in alpha1 of detrended fluctuation analysis (DFA-a1) before (left; i.e., non-fatigued) and after (right; i.e., fatigued) the ramp until voluntary exhaustion from day 1 to day 2. Dots depict individual datapoints, while the boxplots depict the median and interquartile range

In practice, the DFA-a1 value may also be used when individuals are fatigued, and this may alter the reliability. To assess the reliability during fatigued conditions, the participants repeated the low-intensity run post a fatiguing ramp on a second day. Our findings show that both the relative and absolute reliability for HR and VO_2_ were only minimally altered under fatigued conditions, while DFA-a1 showed a lower relative and also absolute reliability (Table 1). For example, the ICC decreased from 0.85 to 0.55 for DFA-a1, and this ICC value in a fatigued state post ramp was lower than the ICC for HR and VO_2_. Since the absolute reliability for DFA-a1 as assessed by SEM decreased only slightly, this suggests that there was a smaller between-individual variability in DFA-a1 values with fatigue, which reduced the relative reliability (ICC) and hereby incorrectly implies a lower overall reliability of DFA-a1 whilst fatigued. This is also confirmed by a smaller between-individual standard deviation for DFA-a1 post-fatigue (Table 1). Although a larger change (~ 8%) in DFA-a1 is required to be confident a real change has occurred post-acute fatigue, as opposed to in non-fatigued conditions (5.5%), the magnitude of the change suggests that the DFA-a1 value may still be useful to monitor autonomous fatigue in fatigued conditions (see also Fig. 2 for the variability within-individuals). In support of this, we previously showed that DFA-a1 was more sensitive to acute fatigue than HR and VO_2_ (Van Hooren et al., 2022). The smaller between-individual variability in DFA-a1 post-fatigue (Fig. 2) suggest that future studies should consider within-individual data to evaluate SEM and SWC values for strengthening a longitudinal monitoring approach (see practical implications). To our knowledge, no previous study has investigated the between-day reliability of DFA-a1 pre and post fatigue making comparison of our findings difficult. For example, a previous study by Boullosa et al. (2014) investigated the within-day reliability of DFA-a1 during walking pre- and post-fatigue, while we compared between-day reliability at similar levels of fatigue.

### Limitations

There are several limitations to this study, as partly discussed previously (Van Hooren et al., 2022). First, in line with previous studies (Gronwald et al., 2021; Rogers et al., 2021a) we had to exclude several individuals from the original sample due to limitations in the wearable technology for accurately determining non-linear HRV indexes. This is an important consideration as it highlights that while the DFA-a1 value is promising, the method may not work consistently for all athletes and in all conditions. Related to this, there was a wide range in the confidence intervals for the ICC values in all variables, which highlights the need for further research using a larger sample size. Second, different HR monitors and variations in belt or ECG lead placement can introduce variations in the DFA-a1 value between and within individuals (Rogers & Gronwald, 2022; Rogers et al., 2022), and this may also impact the reliability. We used the latest generation of the Polar chest belt (Polar H10), which is considered (one of the) most accurate wearable devices to determine HRV in field application and deemed superior to the H7 (Rogers & Gronwald, 2022). Despite this, the current design did not allow us to distinguish the relative impact of technical measurement error, biologic variability and true autonomic nervous system fluctuations on the observed changes between days. Finally, we determined the SWC as 0.5× the CV, yet this is an arbitrary number, and other approaches (e.g., based 0.2× between-individual SD) may yield different SWC thresholds (Franceschini et al., 2023). Nevertheless, we used the SEM and CV instead of a between-individual SD to determine the SWC as the between-individual SD approach is related to group heterogeneity and does not consider variations inherent to repeated measures (Buchheit, 2014).

### Practical Implications

**Fig. 3:**
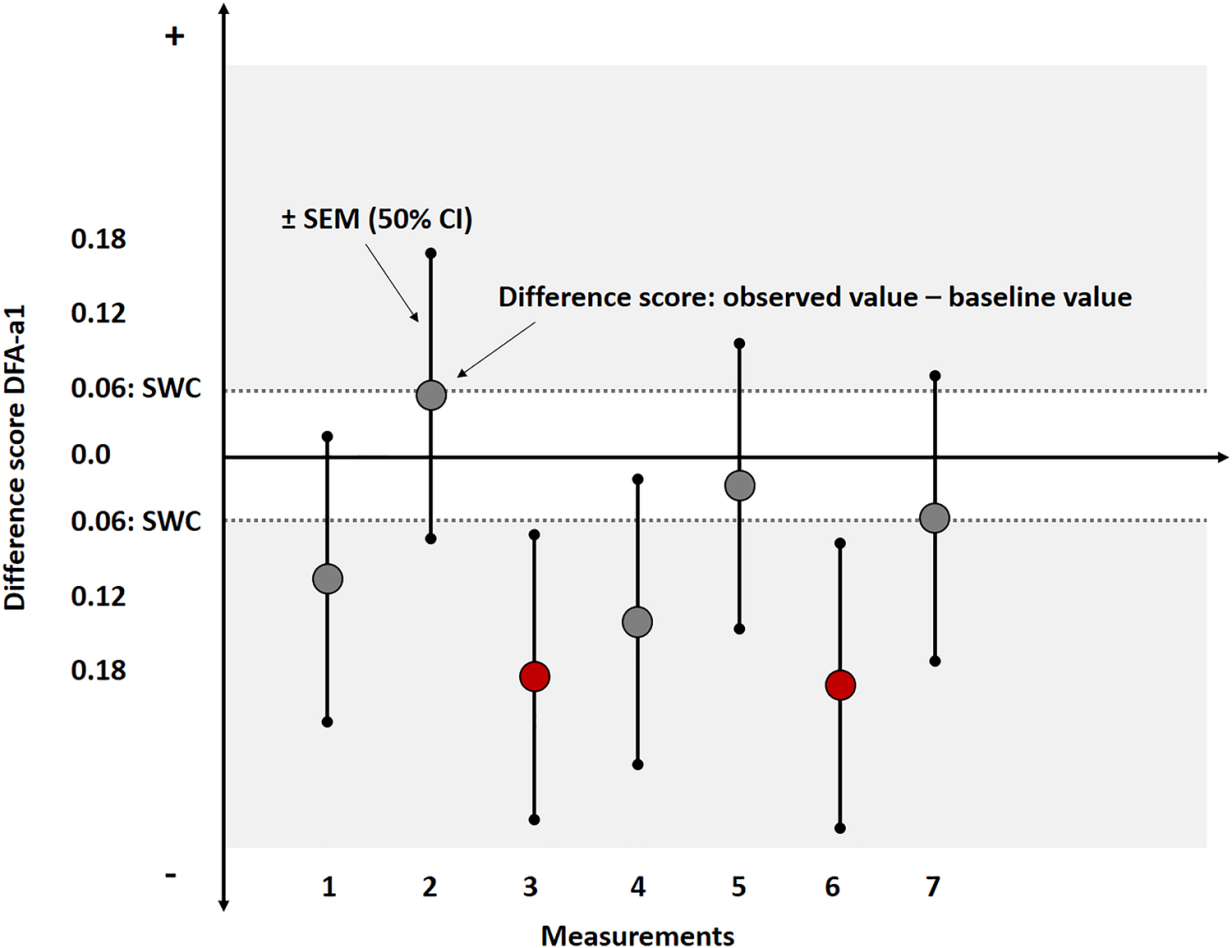
Schematic view of decision-support for the difference score of DFA-a1 (observed value – baseline value) according to the smallest worthwhile change (SWC: 0.06) and the standard error of measurement (SEM: 0.12) during multiple measurements of a single individual in non-fatigued conditions (using the data of the present study); difference scores ± SEM are approximately similar to a 50% confidence interval (CI) (Swinton et al., 2018). In this example, the red marked measurement dots from measurement 3 and 6 show a change deemed worthwhile (decreased DFA-a1 with the SEM not crossing the SWC boundary) which could indicate an adjustment in exercise intensity due to an altered physiological status. Please note: in a longitudinal within-individual approach, a rolling average of regular measurements could be necessary in addition to a consideration of changes in SEM and SWC over time, building an individual normal range for decision-support in combination with psychometrical short scales

The present data show the SWC for DFA-a1 to be 0.06–0.07 in a non-fatigued and fatigued condition, respectively. The SEM was 0.12 and 0.14 in the non-fatigued and fatigued condition, respectively. These values can be used in a simple approach to assist decision-making. Specifically, first a baseline DFA-a1 value (e.g., mean of multiple prior sessions) is subtracted from the observed DFA-a1 value to obtain a change score. Uncertainty (i.e., ± SEM, similar to using 50% CI’s) about the change score is then added to account for measurement error and biological variability, and this is interpreted in relation to the SWC (Fig. 3) (Swinton et al., 2018). With this approach, an athlete performing multiple standardized warm-up sessions would need to exhibit a change in DFA-a1 larger than 0.18–0.21 to be considered as a worthwhile change (Fig. 3). Note that such a change may be considered large relative to the difference between the first and second ventilatory threshold, which is 0.25 (i.e., 07.5 for the first and 0.50 for the second ventilatory threshold). Further data are therefore necessary to analyze whether this threshold is small enough to detect alterations in physiological status due to fatigue. This likely depends on the field of application and on within-individual data in a longitudinal monitoring approach. A large number of values from a single individual may lead to a smaller worthwhile change, which could therefore improve the sensitivity of this method for detecting fatigue and in turn informing on training readiness. Furthermore, additional easily accessible data such as psychometrical questionnaires should be considered as context variables. For example, the Hooper-Index and/or its subitems (i.e., fatigue, muscle soreness, sleep quality, overall stress) have been shown to be promising tools for monitoring applications (Hooper & Mackinnon, 1995; Moalla et al., 2016; Rabbani et al., 2019). Similarly, a combination of both physiological and psychological measures has been shown to be able to identify overtraining (Flockhart et al., 2022).

## Conclusion

Overall, our findings indicate that the DFA-a1 value shows generally good to acceptable reliability and therefore may be useful to monitor an individual’s autonomic balance/physiological status during both non-fatigued and fatigued conditions. Further study of within-individual and intervention data, and the combination with other psychometrical data is necessary to strengthen such an application.

## Data Availability

All data used in analyses are available from the Open Science Framework (https://doi.org/10.17605/OSF.IO/SRVHC).
